# A verified analytical sandwich beam model for soft and hard cores: comparison to existing analytical models and finite element calculations

**DOI:** 10.1007/s00707-023-03497-3

**Published:** 2023-02-22

**Authors:** Juergen Schoeftner

**Affiliations:** Institute of Technical Mechanics, Johannes Kepler University Linz, Altenberger Strasse 69, 4040 Linz, Upper Austria Austria

## Abstract

This paper presents a novel approach of modeling of three-layer beam. Such composites are usually known as sandwich structures if the modulus of elasticity of the core is much smaller than those of the faces. In the present approach, the faces are modeled as Bernoulli–Euler beams, the core as a Timoshenko beam. Taking into account the kinematic and dynamic interface conditions, which means that the perfect bonding assumptions hold for the displacement and each layer is subjected to continuous traction stresses across the interface, a sixth-order differential equation is derived for the bending deflection, and a second-order system for the axial displacement. No restrictions are imposed on the elastic properties of the middle layer, and hence the developed theory also yields accurate results for hard cores. The presented refined theory is compared to analytical models from the literature and to finite element calculations for various benchmark examples. Special focus is laid the boundary conditions and the core stiffness. A parametric study varying the Young modulus of the core shows that the present sandwich model agrees very well with the target solutions obtained from finite element calculations under plane stress assumptions, in particular concerning the transverse deflection, the shear stress distribution and the interfacial normal stress.

## Introduction

Beams and rods made up of composite materials are widely used in mechanical, civil and aerospace engineering. By perfectly bonding various layers with different material properties, one may find a compound with a very high stiffness-to-weight ratio and a composite with a high resistance against a specific load. Even if the single layers are isotropic or orthotropic, the overall behavior of the composite may be anisotropic.

One popular group of these composites is called sandwich structure. These laminates are characterized by a high bending resistance and a very low average density and weight. Usually a relatively soft core is embedded between two stiff skins, which are also called faces. In addition to metallic foam cores, several other possibilities exist to realize soft cores, e.g., truss and lattice cores, honeycomb cores or corrugated cores (see Feng [1] and Ashby [2]). Numerous interesting attributes and further advantages of sandwich structures enlarge the range of engineering applications. Besides the high- strength-to-weight ratio, these advantages include good thermal isolation and protection, acoustic decoupling and energy absorption due to the viscoelastic layer (see Ashby [3]). Many important issues should be considered in the design, analysis, and construction of sandwich structures. Introductions to this field are the well-known books by Zenkert [4], Plantema [5], Marshall [6]. An excellent literature overview on bending, buckling and free vibrations of composite and sandwich beams is presented by Kapania and Racifi [7, 8], Ghugal and Shimpi [9] and Sayyad and Ghugal [10]. Motivated by the remarks in the latter contribution that refined analytical solutions for sandwich structures are rarely available in the scientific literature in comparison with finite element or other numerical solutions, the main focus of this contribution is to close this gap by establishing a modeling procedure that may serve as a starting point for sandwich and laminated beams consisting of orthotropic, anisotropic and piezoelectric properties.

Mead and Markus [11] derived a six-order differential equation for a sandwich beam by neglecting the bending and longitudinal stiffness of the core to calculate resonance frequencies and vibrations of a three-constrained-layer damped beam. Later Mead showed in [12] that Di Taranto [13] had already derived the same outcome for a viscoelastic constraint layer beam. The book by Stamm and Witte [14] gave a very detailed summary of Mead and Markus [11] and Di Taranto [13] model and strongly focused on mathematical modeling of asymmetric sandwich structures. The result is a sixth-order differential equation for the lateral deflection. If the bending stiffness of the skin is not taken into account, one derives a fourth-order differential equation. Only for this case the deflection could be split up into a pure bending deflection of the skin and a shear-dependent deflection.

Heuer [15] derived a fourth-order differential equation for a sandwich beam from d’Alemberts principle. The final equation could be interpreted as the equation of a shear deformable homogeneous beam with effective stiffness if the shear stress is uniformly distributed throughout the laminate depth. In [16], Heuer and Adam investigated a two-layer piezoelectric beam. Interlaminar slip is considered between the layers (the Bernoulli–Euler hypothesis holds for both layers), and it was found that the system is governed by a sixth-order differential equation for the lateral deflection. Adam [17] considered small amplitude vibrations of moderately thick composite plates. Prescribing the continuity of the shear stress according to Hooke’s law a fourth-order differential equation for the plate deflection was derived. In [18], Adam investigated composite plates with eigenstrain, which can be either of thermal, piezoelectric or inelastic nature. It was shown that if Poisson’s ratio and the ratio of mass density and shear modulus of the layers are identical, then the lateral deflection does not depend on the in-plane displacement. Adam and Heuer showed in [19] that the governing equation of a two-layer beam with interfacial slip is similar that of a sandwich beam with thick faces (=anti-sandwich structure). By analogy, it could be shown that the interlaminar slip stiffness is related to the shear modulus and the height of the core. Vo et al.  [20] developed a parabolic shear and normal deformation theory for the bending analysis of functionally graded sandwich beams. Frostig and his co-authors published several works considering the transverse flexibility of the core (see Frostig et al.  [21–24]). Hence, these models can be used to analyze localized pressure and the relative deflection between the skins. Swanson [25] and Swanson and Kim [26] investigated Frostig’s analytical theory to numberotus sandwich problems of interest. The authors agreed that Frostig’s higher-order sandwich model was able to predict high stress concentrations at locations in the surrounding of concentrated loads and supports by comparing the outcome to finite element results. But it was remarked that numerical difficulties may arise from Frostig’s model. A higher-order analysis model for sandwich plates with flexible core is presented by Tian et al. [27]. A simply supported plate was considered and the results for bending deflections and for predicting the natural eigenfrequencies are compared to the Mindlin theory and ANSYS results. Davalos et al. formulate a one-dimensional beam finite element with layer-wise constant shear. Transverse incompressibility was assumed and the constant shear stress from the constitutive relations is transformed into a parabolic stress distribution in a postprocessing step. A modified zig-zag model for analyzing thick composite beams with rectangular cross sections was developed by Icardi [28], which accurately predicted the displacement and stress fields of composite beams. An efficient one-dimensional static model developed for piezoelectric sandwich beams based on third-order zig-zag functions is presented by Kapuria et al. [29].

This work is mainly motivated by the analytical sandwich model from Mead and Markus [11] and Di Taranto [13]. Anyway, it remains unclear, or at least unanswered according to a literature review, why the bending stiffness of the core is neglected a priori in their contributions and when influences on the deflection of a sandwich beam. A minor aim of this contribution is to show the validity range of some analytical models from the literature. The main objective is to present a sandwich model which consists of three isotropic layers, perfectly bonded together, but with different material properties and thicknesses, which also includes three-layer beams with hard cores. On the one hand, the perfect bonding assumptions require the axial and vertical deflections to be equal, on the other hand the stress continuity for the shear stress and the transverse normal stress has to be fulfilled, too. The underlying equations for the core layer are the Timoshenko equations (TS) and for the external layers, which are usually denoted as faces, the Bernoulli–Euler (BE) equations. The final sandwich model is a sixth-order differential equation for the bending deflections, and hence the complexity and mathematical order are equal to the model from Mead and Markus [11]. Benchmark examples with various boundary conditions and core thicknesses are considered and parameter variations concerning the Young modulus of the core are performed and compared to finite element calculations. The range of applicability of the analytical models is discussed, and it is found that the present three-layer modeling approach yields accurate results for both soft and hard cores. The presented modeling procedure can be a starting point for more advanced composite beam models and different material properties (i.e., orthotropic, anisotropic, piezoelectric).

## Recapitulation: equations of motion of a single isotropic layer

**Fig. 1:**
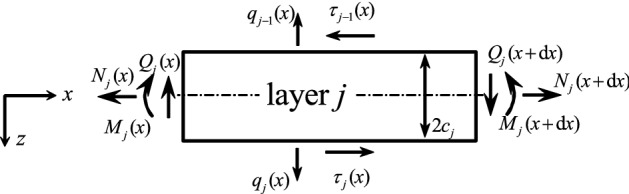
Free body diagram of a beam layer *j* (surface tractions are denoted by $q_{j-1},\, q_{j}$ and $\tau _{j-1},\, \tau _{j}$, the beam forces are $Q_j$, $N_j$ and moment $M_j$)

The basis for the present sandwich model is an accurate beam model taking into account distributed loads that act perpendicular $q_j$ and parallel $\tau _j$ to the upper and lower surfaces. Timoshenko assumptions hold for layer *j* in Fig. 1, and hence the displacement field is

1 $$\begin{aligned} \bar{u}_j (x,z)= & {} {u}_j (x) + z {\psi }_j (x,z)\nonumber \\ \bar{w}_j (x,z)= & {} {w}_j (x) \end{aligned}$$

where ${u}_j (x)$ and ${w}_j (x)$ are the horizontal and the vertical deflection and ${\psi }$ is the rotation angle. The thickness of one layer is $2 c_j$.

From Eq. (1),it follows for the strains

2 $$\begin{aligned} \varepsilon _{xx}= & {} \bar{u}_{j,x} = {u}_{j,x} \nonumber \\ \gamma _{xz} = 2 \varepsilon _{xz}= & {} \bar{u}_{j,z} + \bar{w}_{j,x}= {\psi }_j + {w}_{j,x} \end{aligned}$$

and for the stresses

3 $$\begin{aligned} \sigma _{xx}= & {} E_j \varepsilon _{xx}\nonumber \\ \sigma _{xz}= & {} G_j \gamma _{xz} \end{aligned}$$

Young’s modulus is denoted by $E_j$, for the shear modulus $G_j = E_j/(2+2 \nu _j)$ holds if an isotropic material is assumed. The forces and the bending moment on beam level follow by integration of the stresses. Substituting Eqs. (1) and (2) in Eq. (3), one finds

4 $$\begin{aligned} N_j= & {} \int _{-c_j}^{c_j} \sigma _{xx} b \, \textrm{d}z = \textrm{EA}_j {u}_{j,x} \end{aligned}$$

5 $$\begin{aligned} Q_j= & {} \int _{-c_j}^{c_j} \sigma _{xz} b \, \textrm{d}z = \textrm{GA}^*_j \left( {\psi }_{j} + {w}_{j,x} \right) \end{aligned}$$

6 $$\begin{aligned} M_j= & {} \int _{-c_j}^{c_j} \sigma _{xx} b z \, \textrm{d}z = \textrm{EI}_j {\psi }_{j,x} \end{aligned}$$

Here the axial stiffness, the shear stiffness and the bending stiffness are $$\textrm{EA}_j$$, $$\textrm{GA}^*_j$$ and $$\textrm{EI}_j$$, respectively. The cross-section area is $$A_j = 2 b c_j $$ and the geometrical moment of inertia is $$I_j = 2 b c^3_j/3$$. The star-symbol $$\textrm{G A}^*_j = \kappa G A_j$$ indicates that the shear stiffness also includes a proper shear correction factor $$\kappa $$. The equilibrium conditions can be derived from Fig. 1 and read

7 $$\begin{aligned}{} & {} N_{j,x} + \tau _{j} - \tau _{j-1} = 0 \end{aligned}$$

8 $$\begin{aligned}{} & {} M_{j,x} - Q_j + \left( \tau _{j} + \tau _{j-1} \right) c_j = 0 \end{aligned}$$

9 $$\begin{aligned}{} & {} Q_{j,x} + q_{j} - q_{j-1} = 0 \end{aligned}$$

It is noted that the common relation $M_{j,x} = Q_{j}$ is violated if shear loads over the surfaces are taken into account (see Eq. (8) unless for $\tau _{j-1} = - \tau _{j}$). If unity width is used for the layers $b=1 \, \textrm{m}$, the expressions for the distributed loads coincide with the stress values (e.g., $\sigma _{xz} (x,c) = \tau _{j}/b$, $\sigma _{zz} (x,c) = q_{j}/b$). Substituting the beam forces (4–6) in Eqs. (7–9), one finds three differential equations

10 $$\begin{aligned}{} & {} \textrm{EA}_j {u}_{j,xx} + \tau _{j} - \tau _{j-1} = 0 \end{aligned}$$

11 $$\begin{aligned}{} & {} \textrm{EI}_j {\psi }_{j,xx} - \textrm{GA}^*_j \left( {\psi }_{j} + {w}_{j,x} \right) + \left( \tau _{j} + \tau _{j-1} \right) c_j = 0 \end{aligned}$$

12 $$\begin{aligned}{} & {} \textrm{GA}^*_j \left( {\psi }_{j,x} + {w}_{j,xx} \right) + q_{j} - q_{j-1} = 0 \end{aligned}$$

In case of thin layers the influence of shear on the deflection can be neglected. The Bernoulli–Euler equations follow by differentiating Eq. (11) with respect to *x* and adding to Eq. (12)

13 $$\begin{aligned}{} & {} \textrm{EA}_j {u}_{j,xx} + \tau _{j} - \tau _{j-1} = 0 \nonumber \\{} & {} - EI_j {w}_{j,xxxx} + q_{j} - q_{j-1} + \left( \tau _{j} + \tau _{j-1} \right) _{,x} c_j = 0 \end{aligned}$$

The beam forces and the moment are

14 $$\begin{aligned} N_j^\textrm{BE}= & {} \textrm{EA}_j {u}_{j,x} \end{aligned}$$

15 $$\begin{aligned} Q_j^\textrm{BE}= & {} - \textrm{EI}_j {w}_{j,xxx} + \left( \tau _{j} + \tau _{j-1} \right) c_j \end{aligned}$$

16 $$\begin{aligned} M_j^\textrm{BE}= & {} - \textrm{EI}_j {w}_{i,xx} \end{aligned}$$

Note that the shear force is not proportional to the third derivative of the vertical deflection unless for $\tau _{j-1} = -\tau _{j}$ (see Eq. (15)). This is important to note because the derivations presented by Mead and Markus [11], Di Taranto [13] and Stamm and Witte [14] are erroneous where $M_{j,x} = Q_j$ holds despite the presence of shear tractions.

## Equations of motion of a three-layer beam with soft or hard core

**Fig. 2:**
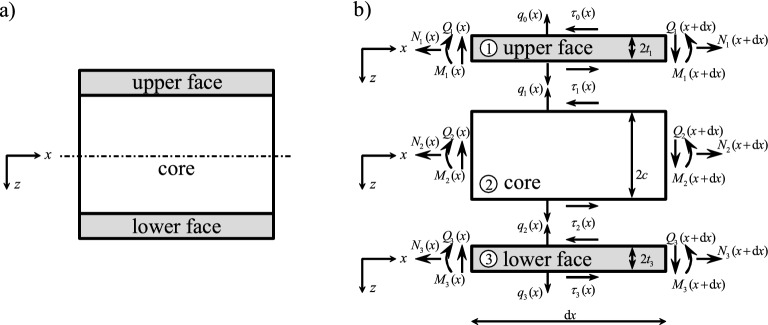
**a** Symmetrical three-layer sandwich beam with surface tractions $q_j$ and $\tau _j$; **b** free-body diagram of an infinitesimal three-layer beam element

In general, the faces (or skins) of a sandwich construction are relatively thin compared to the core thickness, and hence shear effects play a minor rule. Modeling the faces as two BE-beams as in Eq. (13), one finds for the upper (subscript 1) and the lower face (subscript 3) (see also Fig. 2)

17 $$\begin{aligned}{} & {} \textrm{EA}_1 {u}_{1,xx} = \tau _{0} - \tau _{1} \qquad \mathrm {(upper \,\, face)} \end{aligned}$$

18 $$\begin{aligned}{} & {} -\textrm{EI}_1 {w}_{3,xxxx} = q_{0} - q_{1} - \left( \tau _{0,x} + \tau _{1,x} \right) t_1 \end{aligned}$$

19 $$\begin{aligned}{} & {} \textrm{EA}_3 {u}_{3,xx} = \tau _{2} - \tau _{3} \qquad \mathrm {(lower \,\, face)} \end{aligned}$$

20 $$\begin{aligned}{} & {} -\textrm{EI}_3 {w}_{3,xxxx} = q_{2} - q_{3} - \left( \tau _{2,x} + \tau _{3,x} \right) t_3 \end{aligned}$$

The main focus here is to establish a mathematical model for a three-layer beam whose core bending stiffness is not negligible. Considering Timoshenko assumptions, one finds three differential equations for the core from (10–12)

21 $$\begin{aligned}{} & {} \textrm{EA}_2 {u}_{2,xx} = \tau _{1} - \tau _{2} \end{aligned}$$

22 $$\begin{aligned}{} & {} \textrm{EI}_2 {\psi }_{2,xx} - GA^*_2 \left( {\psi _2} + {w}_{2,x} \right) = -\left( \tau _{1} + \tau _{2} \right) c \end{aligned}$$

23 $$\begin{aligned}{} & {} \textrm{GA}^*_2 \left( {\psi }_{2,x} + {w}_{2,xx} \right) = q_{1} - q_{2} \end{aligned}$$

These Eqs. (17–23) contain 11 unknowns (7 degrees of freedom ${w}_{1}, \, {w}_{2}, \, {w}_{3}$, ${u}_{1}, \, {u}_{2}, \, {u}_{3}$, ${\psi _2}$ and 4 interface stresses $q_{1},\, q_{2}, \, \tau _{1},\, \tau _{2}$) which can be computed by 7 differential Eqs. (17–23) and 4 coupling Eqs. (24–26) from the perfect bonding assumptions. The latter read

24 $$\begin{aligned}{} & {} {u}_{1} - t w_{,x} = {u}_{2} - c \psi \end{aligned}$$

25 $$\begin{aligned}{} & {} {u}_{3} + t w_{,x} = {u}_{2} + c \psi \end{aligned}$$

26 $$\begin{aligned}{} & {} {w}_{1} = {w}_{2} = {w}_{3} \end{aligned}$$

A condensed and more comprehensible form of the differential equations is obtained if a symmetrical setup is considered: $$E_1 = E_3$$ and $$t_1 = t_3 \rightarrow EA_f = EA_1 = EA_3 $$, $$EI_f = EI_1 = EI_3 $$. The subscripts 1 and 3 are replaced by the subscript *f* (=face), the subscript 2 is replaced by *c* (=core). In the following, the vertical and horizontal deflections and the rotation angle of the core are denoted by *w*, *u* and $$\psi $$. In a first step the coupling Eqs. (24–26) are substituted in Eqs. (17–23). In a second step, one solves Eqs. (17–20) for the yet unknown interfacial stresses $$q_{1},\, q_{2}, \, \tau _{1},\, \tau _{2}$$ and substitutes the outcome into the remaining three differential Eqs. (21–23)

27 $$\begin{aligned}{} & {} 2 \left( \textrm{EI}_f + \textrm{EA}_f t^2 \right) {w}_{,xxxx} - \textrm{GA}^*_c \left( \psi _{,x} + {w}_{,xx} \right) - 2ct \textrm{EA}_f \psi _{,xxx} \nonumber \\{} & {} = -q_{0} + q_{3} + 2t \left( \tau _{0,x} + \tau _{3,x} \right) \end{aligned}$$

28 $$\begin{aligned}{} & {} \textrm{GA}^*_c \left( \psi + {w}_{,x} \right) - \left( \textrm{EI}_c + 2c^2 \textrm{EA}_f \right) \psi _{,xx} + 2ct \textrm{EA}_f w_{,xxx} = c \left( \tau _{0} + \tau _{3} \right) \end{aligned}$$

29 $$\begin{aligned}{} & {} \left( \textrm{EA}_c + 2 \textrm{EA}_f \right) {u}_{,xx} = \tau _{0} - \tau _{3} \end{aligned}$$

Neglecting the external shear stress $\tau _{0} = \tau _{3} = 0$ one finds

30 $$\begin{aligned}{} & {} 2 \left( \textrm{EI}_f + \textrm{EA}_f t^2 \right) {w}_{,xxxx} - \textrm{GA}^*_c \left( \psi _{,x} + {w}_{,xx} \right) - 2ct \textrm{EA}_f \psi _{,xxx} = - q_{0} (x) \end{aligned}$$

31 $$\begin{aligned}{} & {} \textrm{GA}^*_c \left( \psi + {w}_{,x} \right) - \left( \textrm{EI}_c + 2c^2 \textrm{EA}_f \right) \psi _{,xx} + 2ct \textrm{EA}_f w_{,xxx} = 0 \end{aligned}$$

32 $$\begin{aligned}{} & {} \left( \textrm{EA}_c + 2 \textrm{EA}_f \right) {u}_{,xx} = 0 \end{aligned}$$

The solution for the decoupled axial displacement (32) reads

33 $$\begin{aligned} u (x) = A_0 + A_1 x \end{aligned}$$

The two unknowns $A_0, \, A_1$ can be determined by corresponding kinematic or dynamic boundary conditions. One observes that the axial displacement of the middle axis of the core is zero (unless normal forces for the boundary conditions are considered).

Integrating Eq. (30) and adding the results to Eq. (31) one finds for the rotation angle

34 $$\begin{aligned} \psi = \frac{ \left( 2 \textrm{EI}_f + 2 t (c+t) \textrm{EA}_f \right) w_{,x} + \int \int \int q_0(x) \, \textrm{d}x^3 }{ \textrm{EI}_c + 2c (c+t) \textrm{EA}_f } + B_1 + B_2 x + B_3 x^2 \end{aligned}$$

Inserting Eq. (34) into Eq. (31) and integrating the resulting equation yields the second-order differential equation for the deflection

35 $$\begin{aligned} P w(x) - Q w_{,xx} = f(x) \end{aligned}$$

where $P,\, Q$ and the inhomogeneous term *f*(*x*) read

36 $$\begin{aligned} P= & {} \frac{ \textrm{GA}_c^* \left[ \textrm{EI}_c + 2 \left( \textrm{EI}_f + \textrm{EA}_f (c+t)^2 \right) \right] }{ \textrm{EI}_c + 2c (c+t) \textrm{EA}_f }\nonumber \\ Q= & {} \frac{ 4 c^2 \textrm{EI}_f \textrm{EA}_f + 2 \textrm{EI}_c \left( \textrm{EI}_f + \textrm{EA}_f t^2 \right) }{ \textrm{EI}_c + 2c (c+t) \textrm{EA}_f } \end{aligned}$$

37 $$\begin{aligned} f(x)= & {} - B_4 - B_1 \textrm{GA}_c^* x - B_2 \textrm{GA}_c^* \frac{ x^2 }{2} + B_3 \frac{ (6 \textrm{EI}_c + 12 c^2 \textrm{EA}_f)x - \textrm{GA}_c^* x^3 }{ 3 } \nonumber \\{} & {} \quad - \frac{ \textrm{GA}_c^* }{ \textrm{EI}_c + 2c (c+t) \textrm{EA}_f } \int \int \int \int q_0(x) \, \textrm{d}x^4 + \frac{ 2c^2 \textrm{EA}_f + \textrm{EI}_c }{ \textrm{EI}_c + 2c (c+t) \textrm{EA}_f } \int \int q_0(x) \, \textrm{d}x^2 \end{aligned}$$

From a mathematical point of view, the three-layer sandwich model is reduced to a second-order differential Eq. (36), which holds for an arbitrary distributed load $q_0(x)$. The eigenvalues of the system are given by Eq. (40). Six integration constants must be satisfied: four constants $({B_1,\,B_2,\,B_3,\,B_4})$ are already included in *f*(*x*) (see Eq. (37)): two additional ones occur from the second-order differential equation (see Eq. (35)).

In order to provide a more detailed relation to other sandwich models (see sections A.1 and A.2) and for reasons of an easy implementation in symbolic computational software like MATHEMATICA, it is more appropriate to convert Eq. (35) into a sixth-order differential equation

38 $$\begin{aligned} R \frac{\partial ^4 w(x)}{\partial x^4} - S \frac{\partial ^6 w(x)}{\partial x^6} = -\textrm{GA}_c^* q_0(x) + \left( 2c^2 \textrm{EA}_f + \textrm{EI}_c \right) q_{0,xx}(x) \end{aligned}$$

where the constants *R* and *S* read

39 $$\begin{aligned} R= & {} \textrm{GA}_c^* \left[ \textrm{EI}_c + 2 \left( \textrm{EI}_f + \textrm{EA}_f (c+t)^2 \right) \right] \nonumber \\ S= & {} 4 c^2 \textrm{EI}_f \textrm{EA}_f + 2 \textrm{EI}_c \left( \textrm{EI}_f + \textrm{EA}_f t^2 \right) \end{aligned}$$

Equation (38) is a sixth-order differential equation for the deflection *w* where four eigenvalues are zero, the remaining nontrivial eigenvalues are the root of

40 $$\begin{aligned} \alpha ^2 = \frac{ GA_c^* \left[ \textrm{EI}_c + 2 \left( \textrm{EI}_f +\textrm{EA}_f (c+t)^2 \right) \right] }{ 4c^2 \textrm{EI}_f \textrm{EA}_f + 2 \textrm{EI}_c \left( \textrm{EI}_f + \textrm{EA}_f t^2 \right) } \end{aligned}$$

The solution for the deflection is

41 $$\begin{aligned} w (x) = w_{j}(x) + \sum _{i=1}^4 B_i x^{(i-1)} + B_5 \textrm{exp} \left( -\alpha x \right) + B_6 \textrm{exp} \left( \alpha x \right) \end{aligned}$$

where $w_{j}(x)$ is the inhomogeneous solution. The constants $B_1-B_6$ must be determined from the boundary conditions (see Sect. 3.1).

### Boundary conditions

**Fig. 3:**
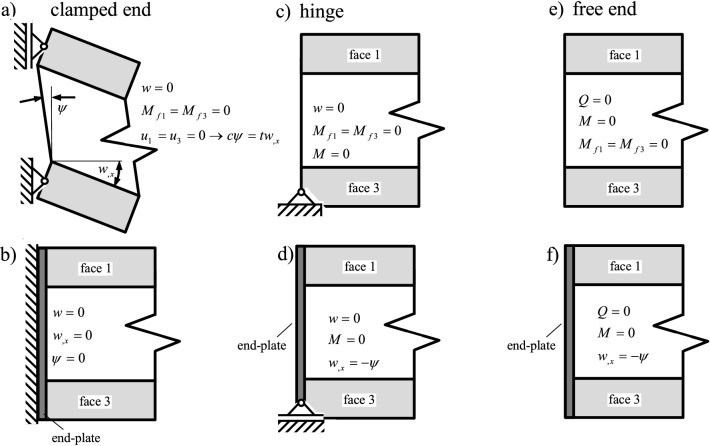
Several boundary conditions (clamped with and without end plate **a**, **b**; soft and hard hinged support **c**, **d**; free end with and without end plate **e**, **f**)

As opposed to the classical beam theory, where four boundary conditions must be prescribed in order to calculate the constants from integration, the sixth-order differential equation for the three-layer beam requires three boundary conditions on the left and on the right side of the beam (note: the boundary conditions for the axial deflection *u*(*x*) (see Eq. (33)), are not discussed here, because external shear tractions and normal forces are not considered, hence $A_0 =A_1 = 0$ holds). In the following, a clamped end, a hinge and a free end are discussed. For each boundary, two possibilities exist depending if a rigid end plate is attached or not (see also the extensive discussion in Stamm and Witte [14] for Mead and Markus’ sandwich model [11]). It is clear from Fig. 3 that an end plate is an additional kinematic constraint causing the core and face angles to be equal $\psi = -w_{,x}$.

According to Eqs. (17), (19), (24) and (25) the interfacial shear stresses read in case of a a symmetrical sandwich panel and a vanishing axial displacement of the core $u=0$

42 $$\begin{aligned} \tau _{1} = \tau _{2} = \textrm{EA}_f \left( c \psi _{,x} - t w_{,xx} \right) \end{aligned}$$

The following relations hold between the kinematic degrees of freedom and the beam forces of the core and the face:

- normal force:
  43 $$\begin{aligned} N_{f}= & {} \textrm{EA}_f u_{1,x} = \textrm{EA}_f \left( - c \psi _{,x} + t w_{,xx} \right) \nonumber \\= & {} \textrm{EA}_f u_{3,x} = \textrm{EA}_f \left( c \psi _{,x} - t w_{,xx} \right) \end{aligned}$$
  44 $$\begin{aligned} N_{c}= & {} \textrm{EA}_c u_{,x} = 0 \end{aligned}$$
  45 $$\begin{aligned} N= & {} N_{c} + 2 N_{f} \end{aligned}$$
- shear force:
  46 $$\begin{aligned} Q_c&= {} \textrm{GA}_c^* \left( \psi + w_{,x} \right) \nonumber \\ Q_{f}&= {} -\textrm{EI}_f w_{,xxx} + \tau _{1} t\nonumber \\&= {} -\textrm{EI}_f w_{,xxx} + \tau _{2} t \end{aligned}$$
  47 $$\begin{aligned} Q&= {} Q_{c} + 2 Q_{f} \end{aligned}$$
- bending moment:
  48 $$\begin{aligned} M_c= & {} \textrm{EI}_c \psi _{,x} \end{aligned}$$
  49 $$\begin{aligned} M_{f}= & {} -\textrm{EI}_f w_{,xx} \end{aligned}$$
  50 $$\begin{aligned} M= & {} M_c + 2 M_f + 2 N_{f} \left( c+t \right) \end{aligned}$$

#### Clamped boundary: with and without rigid end plate

Figure 3a, b shows a clamped boundary with and without rigid end plate.

Considering an end plate means that none of the kinematic degrees of freedom can move at $x^* = 0$

51 $$\begin{aligned} w (x^*) = 0\nonumber \\\quad w_{,x} (x^*) = 0\nonumber \\\quad \psi (x^*) = 0 \end{aligned}$$

Another possibility is if the upper and the lower face are fixed by two hinges. The bending moment of the face will vanish $M_f (x^*) = 0$ and from Fig. 3a it becomes clear that the rotation angle of the core will be nonzero, i.e., $\psi (x^*) \ne 0$, Eqs. (24) and (25) describe the kinematic constraints between deflection *w* and $\psi$

52 $$\begin{aligned} w (x^*) = 0\nonumber \\\quad M_f (x^*) = 0 \rightarrow \textrm{EI}_f w_{,xx} (x^*) = 0\nonumber \\\quad u_1 (x^*) = u_3 (x^*) = 0 \rightarrow c \psi (x^*) = t w_{,x} (x^*) \end{aligned}$$

#### Hinged boundary: with and without end plate

Figure 3c, d shows two possibilities for a hinged boundary. In case of a soft hinged support (without rigid end plate) the bending moment of the face $M_f$, the total bending moment *M* and the deflection *w* vanish

53 $$\begin{aligned} w (x^*) = 0\nonumber \\\quad M (x^*) = 0\nonumber \\\quad M_f (x^*) = 0 \end{aligned}$$

In case of a hard hinged support (with rigid end plate), the face bending moment condition must be replaced by the kinematic constraint Eq. (24)

54 $$\begin{aligned} w (x^*) = 0\nonumber \\\quad M (x^*) = 0\nonumber \\\quad \psi (x^*) = -w_{,x} (x^*) \end{aligned}$$

#### Free end: with and without end plate

If all of the vertical faces of the layers are free, the dynamic boundaries are free

55 $$\begin{aligned} M (x^*) = 0\nonumber \\\quad M_f (x^*) = 0 \nonumber \\\quad Q (x^*) = 0 \end{aligned}$$

If an end plate is present, then

56 $$\begin{aligned} M (x^*) = 0\nonumber \\ \quad Q (x^*) = 0\nonumber \\ \quad \psi (x^*) = -w_{,x} (x^*) \end{aligned}$$

hold.

## Examples: verification of the presented sandwich model

In this section, the presented refined sandwich theory (RSWT) is compared to both Mead–Markus models: the $6{\textrm{th}}$-order model (see Appendix A.1) does not include the core bending stiffness, the simplified $4{\textrm{th}}$-order model (see Appendix A.2) also disregards the face bending stiffness. It becomes clear that the latter one cannot satisfy all boundary conditions (see also Stamm and Witte [14] by comparing p.25–p.90). The results from the two-dimensional finite element calculations under plane stress assumptions serve as target solutions. Additionally, the outcome of an equivalent single layer model ($\textrm{ESL}_\textrm{Timo}$) taking into account shear deformation (Timoshenko assumption) is computed (Appendix C). For the variations concerning the core elasticity (see Figs. 5, 8 and 10), results are also computed for a structure with totally negligible core stiffness ($\textrm{BE}_\textrm{face}$, i.e., the structure consists of perfectly slipping faces without interfacial shear stress, and hence for the core $\textrm{GA}_c^* \approx \textrm{EI}_c \approx 0$ holds, Appendix D). These last two models are limiting cases ($\textrm{ESL}_\textrm{Timo}$ and $\textrm{BE}_\textrm{face}$) and should not be understood as specific sandwich models. The only intention of these models is to show their very limited validity range: If the Young modulus of the core and of the faces are of the same order, the results should be very close to $\textrm{ESL}_\textrm{Timo}$; if the core is very soft, the results from $\textrm{BE}_\textrm{face}$ should be obtained.

The vertical load on the upper face is uniformly distributed $q_0 (x) = 1 \, \mathrm {N/m}$ over the length $l=1 \, {\textrm{m}}$. As benchmark examples a cantilever and a clamped-hinged sandwich beam are considered and the lateral deflections are shown. The shear stress distribution in thickness direction $\sigma _{xz}(x,z)$ and the interfacial normal stress (i.e., $\sigma _{zz}(x,c) = q_2(x)/b$ and $\sigma _{zz}(x,-c) = q_1(x)/b$ (see Fig. 2)) are computed for the cantilever sandwich. The thickness-to-length ratio is $\lambda _t = (2c+4t)/l = 1/8$ for all benchmark examples, the relative thickness of the core $\lambda _c = 2c/(2c+4t)$ (ratio of core thickness to total thickness) is either 0.2 or 0.8.

For the elasticity of the core, the non-dimensional variable $\mu = E_c / E_f = G_c/ G_f$ (ratio of Young’s and shear moduli) is introduced. From a practical point of view, parameter variations of the relative core elasticity immediately show the validity range and accuracy of each analytical model under investigation. The following abbreviations are used for the analytical models:

- RSWT (refined sandwich theory): governed by the $6{\textrm{th}}$-order differential equations (30) and (31) taking into account the core bending stiffness (see Sect. 3.
- $\textrm{MM}_{6{\textrm{th}}}$: Mead–Markus [11] or Di Taranto [13] sandwich model disregarding the core bending stiffness, see Appendix A.1 and Stamm and Witte [14] p.86.
- $\textrm{MM}_{4{\textrm{th}}}$: Mead–Markus [11] or Di Taranto [13] sandwich model disregarding the core and the face bending stiffness, see Appendix A.2 and Stamm and Witte [14] p.22.
- $\textrm{ESL}_{\textrm{Timo}}$: equivalent single layer theory with Timoshenko assumption, see Appendix C
- $\textrm{BE}_\textrm{face}$: the core bending and shear stiffness is totally neglected. The structure is reduced to a sliding two-layer beam with vanishing interfacial shear stress $\tau _1 = \tau _2 = 0$, see Appendix D.

The geometrical and material parameters for the core and the faces of the sandwich beam are summarized in Table 1. Special attention must be paid to the choice of the shear correction factor for the core layer. For typical sandwich structures, when the elasticity of the core is small, the shear stress distribution of the core will be constant or at least very close to a constant distribution, and hence setting $\kappa = 1$ is justified. As the results from Fig. 6 and the comparison to FE results show this is also a reasonable choice for moderately soft cores, i.e., if $\mu = 1/10$.

**Table 1:** Parameters for the numerical examples

Variable (unit)	Value	Description
$l \,\quad (\textrm{m})$	0.8	Length
$\lambda _t = (2c + 4t)/l \,\quad (\mathrm {-})$	1/8	Thickness-to-length ratio
$E_f \,\quad (\textrm{Nm}^{-2})$	$2.1 \times 10^{11}$	Young’s modulus of face
$\nu _c = \nu _f \,\quad (\mathrm {-})$	0.3	Poisson ratio of core and face
$\kappa = 1 \,\quad (\mathrm {-})$	1	Shear correction factor of core
$\lambda _c = 2c/(2c + 4t) \,\quad (\mathrm {-})$	(varies)	Core thickness ratio
$c = \lambda _c \lambda _t l/2 \,\quad (\textrm{m})$	(varies)	Half of the core thickness
$t = (1-\lambda _c) \lambda _t l/4 \,\quad (\textrm{m})$	(varies)	Half of the face thickness
$E_c = E_f \mu \,\quad (\textrm{Nm}^{-2})$	(varies)	Young’s modulus of core
$G_f = E_f/(2+2\nu _f) \,\quad (\textrm{Nm}^{-2})$	(varies)	Shear modulus of face
$G_c = E_c/(2+2\nu _c) \,\quad (\textrm{Nm}^{-2})$	(varies)	Shear modulus of core
$A_f = 2 b t \,\quad (\textrm{m}^2)$	(varies)	Cross-section area of face
$A_c = 2 b c \,\quad (\textrm{m}^2)$	(varies)	Cross section area of core
$I_f = 2 b t^3/3 \,\quad (\textrm{m}^4)$	(varies)	Geometric moment of inertia of face
$I_c = 2 b c^3/3 \,\quad (\textrm{m}^4)$	(varies)	Geometric moment of inertia of core

### Clamped (with rigid end plate): free (without rigid end plate)

The first example is a cantilever sandwich beam with a thickness-to-length ratio $\lambda _t = 1/8$. The core thickness is $0.08 \, \textrm{m}$, the thickness of each face is $0.01 \, \textrm{m}$, and hence $\lambda _c = 0.8$ follows from Table 1.

**Fig. 4:**
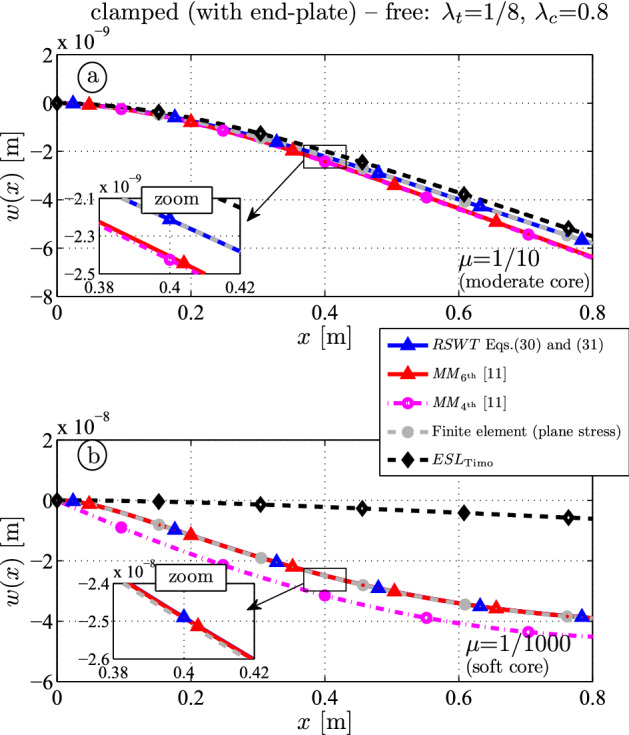
Deflection for a clamped-free sandwich beam with thick core $\lambda _c = 0.8$ (hard core $\mu = 1/10$ (**a**); soft core $\mu = 1/1000$ (**b**))

Figure 4a, b show the vertical deflection for a (relatively) hard ($\mu = E_c/E_f = 1/10$) and a soft core ($\mu = E_c/E_f = 1/1000$), respectively. All analytical theories show similar results (Fig. 4a). But the zoom figure reveals that the deflection from the FE (gray) and the present sandwich theory (blue) is $-2.21 \times 10^{-9} \, \textrm{m}$ at $x=l/2$. The $4{\textrm{th}}$ (magenta) and $6{\textrm{th}}$ (red)-order models of Mead–Markus overestimate the outcome $-2.41 \times 10^{-9} \, \textrm{m}$ and $-2.42 \times 10^{-9} \, \textrm{m}$, respectively, because the bending stiffness of the core is neglected. The ESL-theory (black) is a little too stiff and underestimates the result $-1.99 \times 10^{-9} \, \textrm{m}$.

If the core is weaker (Fig. 4b), *RSWT* (blue) and $MM_{6{\textrm{th}}}$ (red) almost coincide $-2.49 \times 10^{-8} \, \textrm{m}$ and both results are close to the the numerical outcome $-2.50 \times 10^{-8} \, \textrm{m}$ (gray) at $x=l/2$. $MM_{4{\textrm{th}}}$ overestimates the outcome: the clamped boundary is inaccurately modeled because the bending stiffness of the faces are neglected. As expected the ESL (black) yields much too stiff results for the soft core example.

**Fig. 5:**
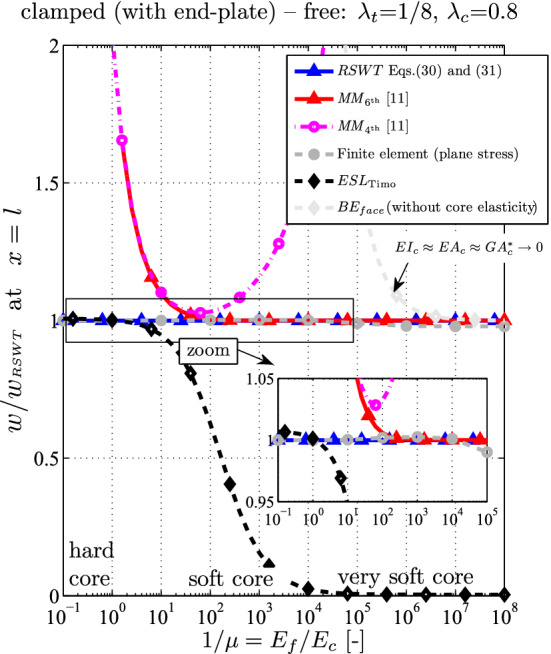
Relative difference of the tip deflection as a function of the core elasticity $\mu = E_c / E_f$

A good overview of the validity range for all the analytical theories shows Fig. 5. The relative tip deflection is shown (normalized by the RSWT outcome $w_{RSWT} (l)$) over the variation of the Young and the shear modulus of the core. The non-dimensional variable is $\mu = E_c/E_f = G_c / G_f$. The Timoshenko *ESL*-beam (black) yields reliable results for $\mu > 1/10$ when the error is below $5 \%$. When the core bending stiffness becomes less important (i.e., for $\mu < 1/50$) the results from both Mead–Markus models are accurate. It can be seen that the $6{\textrm{th}}$-order model converges to the presented RSWT results. Contrary, the $4{\textrm{th}}$-order MM model should be used only for $1/200> \mu > 1/10$. It is noted that compressibility effects of the core cannot be considered by any of the analytical models. The finite element results includes such effects, of course, but they play a minor role even for extremely soft cores: at $\mu = 10^{-7}$ the thickness deformation is only $0.98\%$ of the vertical deflection.

**Fig. 6:**
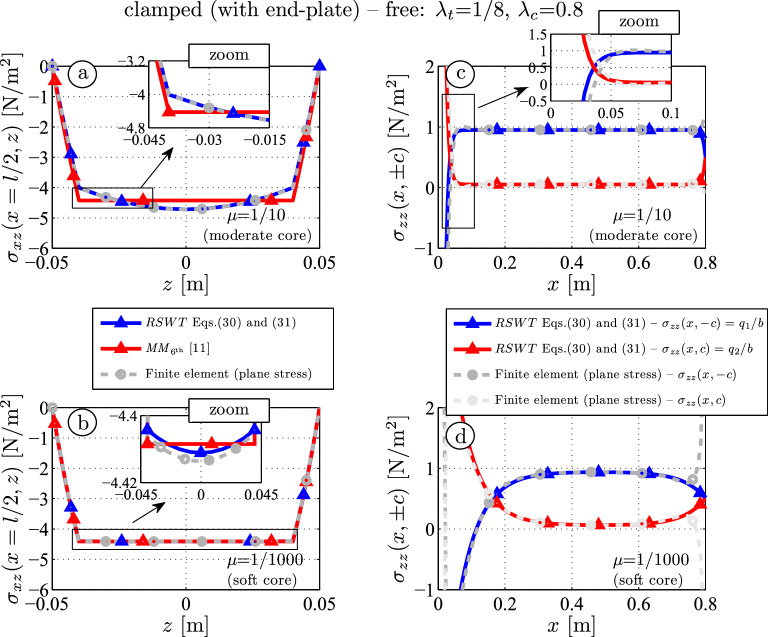
Shear stress distribution (**a**, **b**) and interfacial normal stress (**c**, **d**) for moderate and soft cores

Figure 6a, b shows the shear stress distribution at $x=l/2$ for $\mu = 1/10$ and $\mu = 1/1000$. One can recalculate the shear stress of each layer in some simple post-processing steps: assuming a quadratic shear stress of the form $\sigma _{xz} = C_0 +C_1z+C_2z^2$ for the core and computing $\tau _1$, $\tau _2$ from Eq. (42) and the shear force $Q_c$, the three unknowns $C_0,\,C_1,\,C_2$ are determined. For the faces the same procedure can be done in an analogous manner.

One observes a perfect fit between the finite element and the RSWT results: The parabolic part of the core vanishes for the *MM* model by definition because normal stress is neglected. The shear stress distribution in the faces is almost linear: According to the balance of linear momentum $\sigma _{xx,x}+\sigma _{xz,z}=0$, the linear shear stress contribution is caused by the constant axial stress due to the axial force $N_f$, the quadratic part is caused by the linear axial stress distribution from the face bending moment $M_f$. It is noted that the quadratic term is very small if the faces are thin, see Fig. 6b.

The interfacial normal stresses $\sigma _{zz}(x,\pm c)$, which equal $q_1$ and $q_2$ for beam of unit width, is shown in Fig. 6c, d. At the upper face the normal stress is close to one (*FE*: $0.950\, \textrm{Nm}^{-2}$, *RSWT*: $0.948\, \textrm{Nm}^{-2}$ at $x=l/2$, Fig. 6c). Only close to the clamped and the free end where some end effects occur the *FE* and *RSWT* differ.

In case of a softer core (Fig. 6d), the presented theory also agrees with the numerical result. It is noted that the original paper of Mead–Markus [11] does not give any hints how to calculate the interfacial normal stress, so it is omitted here.

### Clamped (with rigid end plate): soft hinged support

Next a clamped-hinged sandwich panel is investigated. Again the deflection curves with moderately hard and soft core are plotted and the thickness-to-length ratio is $\lambda _t = 1/8$. Parameter variations of the core stiffness show the relative difference to the outcome from RSWT. Section 4.2.1 shows the results for a thick core $\lambda _c = 0.8$ (sandwich); Sect. 4.2.2 shows the results for a thin core $\lambda _c = 0.2$ (anti-sandwich).

#### Thick core ($\lambda _c = 0.8$)

**Fig. 7:**
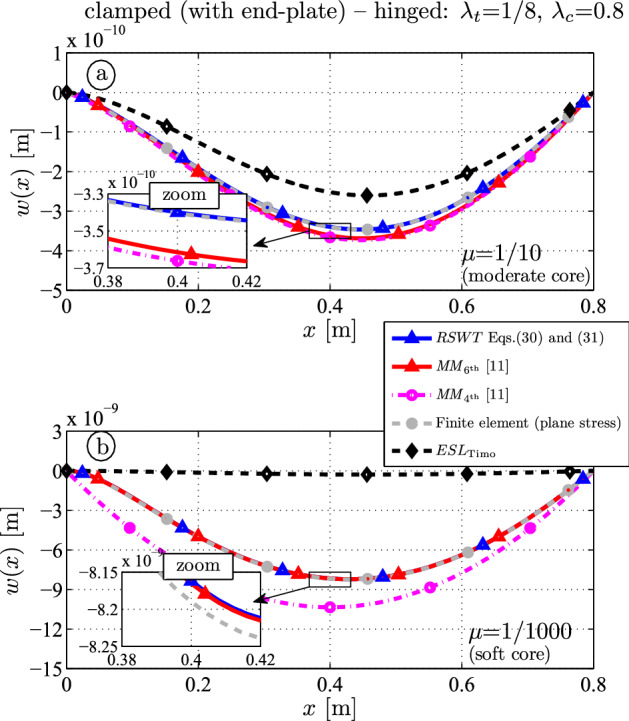
Deflection for a clamped-hinged sandwich beam ($\lambda _t = 1/8$, $\lambda _c = 0.8$): **a** hard core ($\mu = 1/10$); **b** soft core ($\mu = 1/1000$)

The core thickness is $0.08 \, \textrm{m}$; the thickness of each face is $0.01 \, \textrm{m}$. The deflection is shown in Fig. 7. For $\mu = 1/10$ (Fig. 7a), one observes that the FE outcome (gray) matches with the present theory (blue). At $x=l/2$ the deflections are $-3.41 \times 10^{-10} \, \textrm{m}$ (FE) and $-3.40 \times 10^{-10} \, \textrm{m}$ (RSWT). $MM_{6{\textrm{th}}}$ (red) and $MM_{4{\textrm{th}}}$ (magenta) overshoot the results ($-3.62 \times 10^{-10} \, \textrm{m}$ and $-3.66 \times 10^{-10} \, \textrm{m}$, respectively).

For soft cores (Fig. 7b), $MM_{6{\textrm{th}}}$ (red) is close to RSWT (blue), and the deflections are $-8.17 \times 10^{-10} \, \textrm{m}$ at $x=l/2$. The target solution (FE gray) is $-8.19 \times 10^{-10} \, \textrm{m}$. As for the cantilever beam (Fig. 4) $MM_{4{\textrm{th}}}$ (magenta) overestimates the outcome mainly due to improper modeling of the clamped boundary condition at $x=0$.

**Fig. 8:**
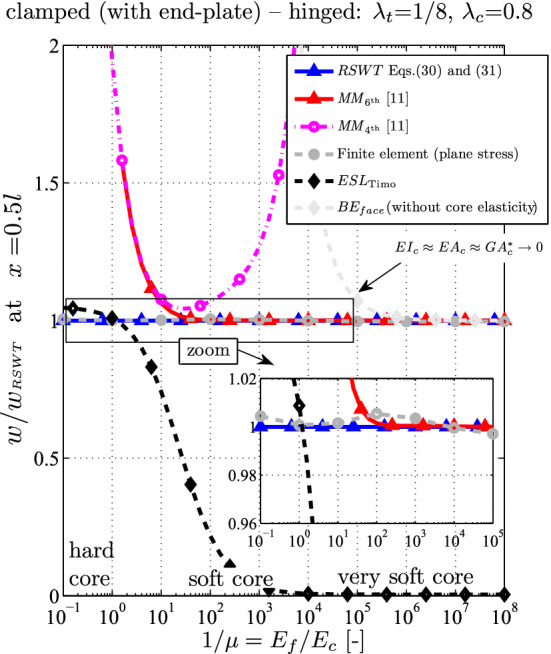
Relative difference of the midspan deflection for a clamped-hinged sandwich beam ($\lambda _t = 1/8$, $\lambda _c = 0.8$ as a function of the core elasticity $\mu = E_c / E_f$)

The relative difference of the deflection at $x=l/2$ over the core elasticity is shown in Fig. 8. The tendency and validity ranges of the various analytical theories are very similar as in the previous section for the cantilever: The ESL should be used only for hard cores, i.e., $\mu > 1/5$, while the assumption of a vanishing axial core stress ($MM_{6{\textrm{th}}}$) is accurate for $\mu < 1/10$. Neglecting both the bending stiffness of the core and of the faces ($MM_{4{\textrm{th}}}$) yield unrealistic results unless for the short interval $1/100< \mu < 1/10$ when the error is below $5\%$. It is noted that the incompressibility assumption holds for the whole range: the relative thickness deformation is $0.3 \%$ at $\mu = 10^{-5}$ in the FE model.

#### Thin core ($\lambda _c = 0.2$)

Now the core thickness of the clamped-hinged anti-sandwich beam is $0.02 \, \textrm{m}$, but the thickness of each face is $0.04 \, \textrm{m}$.

**Fig. 9:**
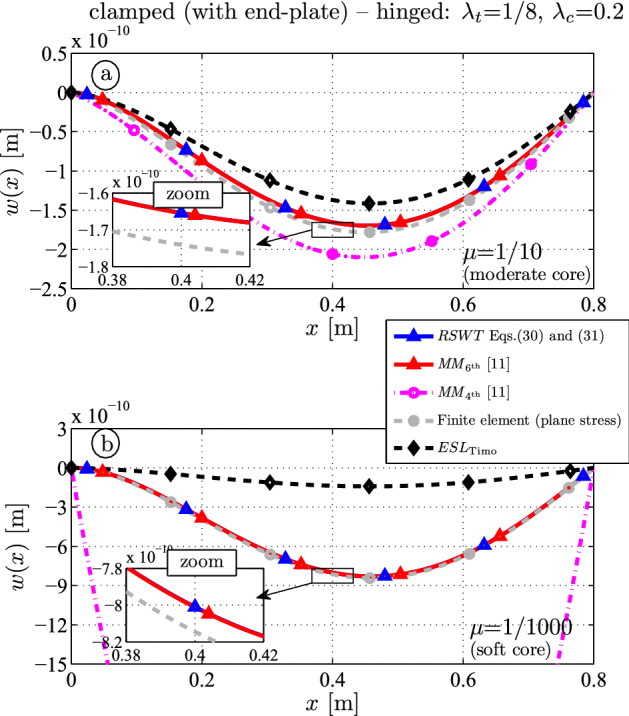
Deflection for a clamped-hinged sandwich beam ($\lambda _t = 1/8$, $\lambda _c = 0.2$): **a** hard core ($\mu = 1/10$); **b** soft core ($\mu = 1/1000$)

The bending stiffness of the core is small, and hence the $MM_{6{\textrm{th}}}$ (red) and the RSWT outcome (blue) are almost identical: $-1.66 \times 10^{-10} \, \textrm{m}$ at $x=l/2$ see Fig. 9a for $\mu = 1/10$. The target solution from FE (gray, $-1.74 \times 10^{-10} \, \textrm{m}$) is underestimated because both theories assume infinite shear stiffness of the faces due to the Bernoulli–Euler assumption. This can be explained as follows: The beam length is $l=0.8 \, \textrm{m}$, so the face thickness is $0.04 \, \textrm{m}$ which corresponds to a thickness-to-length ratio 1/20 and the influence of shear on the deflection becomes more dominant than in Sect. 4.2.1. Without going into detail, $MM_{4{\textrm{th}}}$ (magenta) overshoots and *ESL* (black) undershoots the outcome.

If the core is less stiff (Fig. 9b, $\mu = 1/1000$), both $6{\textrm{th}}$-order theories of course match again, but the difference to the FE results remains due to the infinite shear rigidity of the faces as before.

**Fig. 10:**
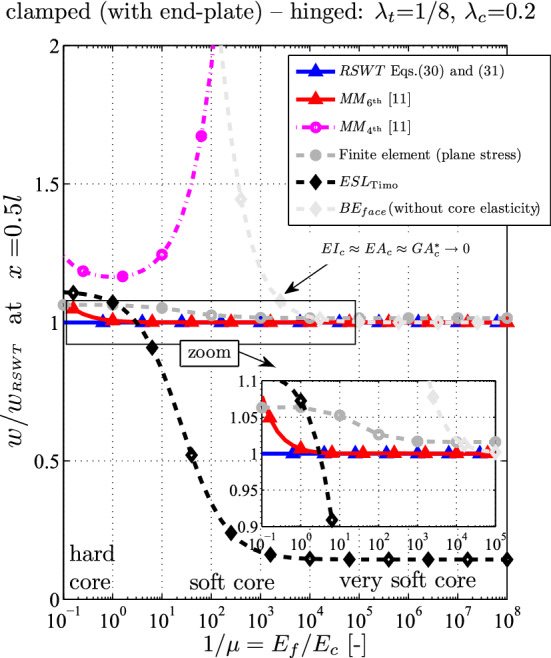
Relative difference of the midspan deflection for a clamped-hinged sandwich beam ($\lambda _t = 1/8$, $\lambda _c = 0.2$ as a function of the core elasticity $\mu = E_c / E_f$)

One observes from Fig. 10 that FE (gray), RSWT (blue) and $MM_{6{\textrm{th}}}$ (red) are in good agreement. If the core is harder, the no-bending-stiffness assumption from the latter theory compensates the Bernoulli–Euler assumption for the faces. For softer cores $\mu < 1$ its results converge to the RSWT as expected. For $\mu = 1/10$ both analytical results underestimate the FE outcome by $5.3\%$. For $\mu < 1/10^5$ the difference is $1.6\%$ and remains constant because of the infinite shear rigidity of the faces. The ${4{\textrm{th}}}$-order Mead–Markus theory yields inaccurate results independent of the core’s Young modulus and should be avoided for anti-sandwich beams.

## Conclusion

In this contribution, a novel approach of modeling a three-layer beam is presented. This type of composite includes a sandwich beam as representative example. The middle layer is modeled as a Timoshenko beam subjected to traction forces parallel and perpendicular to its upper and lower surfaces. The faces, also denoted as constraining layers, are modeled as Bernoulli–Euler beams. Eliminating the interfacial stresses between the middle and its attached layers and considering the perfect bonding assumptions, a sixth-order differential equation for the bending deflections is derived. The outcome is an extension of the sixth-order sandwich theory from Di Taranto and Mead-Markus but it includes also the bending stiffness of the core layer although the mathematical complexity remains the same. Hence, the presented analytical theory also holds for both soft and hard cores and can be extended to find suitable models for arbitrary composite layouts with different layer properties. Finally, the presented refined theory is compared to analytical models from the literature and to finite element calculations for various benchmark examples. Different boundary conditions and core stiffness (by varying its thickness and Young’s and shear moduli) are considered showing that the derived theory agrees best with the target results from finite element analysis.

## Figures and Tables

**Fig. 11:**
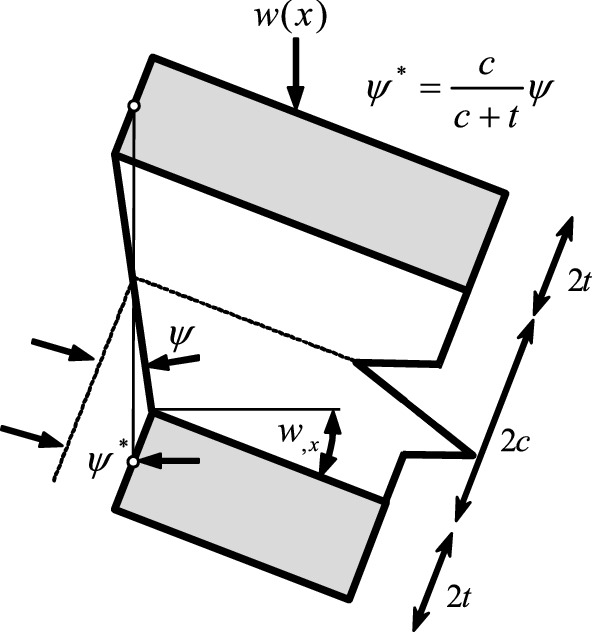
Deformed sandwich element according to Mead–Markus [11] and Di Taranto [13] (denoted as $MM_{4\textrm{th}}$ and $MM_{6\textrm{th}}$)

**Fig. 12:**
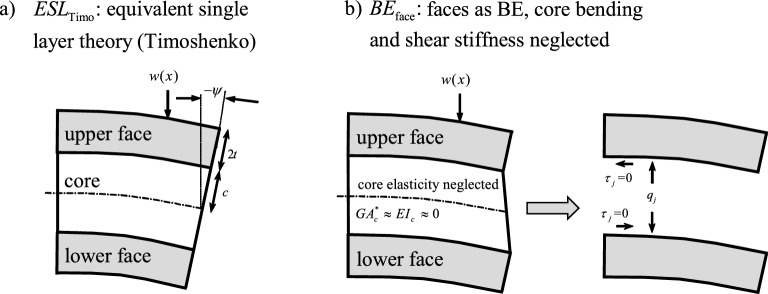
**a** Equivalent single layer theory and **b** without core elasticity

## Appendix A Sandwich theory according to Mead–Markus [11] and Di Taranto [13]

In the following, the sandwich equations from Mead–Markus [11] and Di Taranto [13] are summarized. Their theory is limited to relatively soft cores because of $E_c \approx 0$ (i.e., the longitudinal and the bending stiffness of the core are neglected).

### A.1 Sixth-order sandwich theory

Stamm and Witte [14] present a more comprehensive derivation and the find the following differential equations

A1 $$\begin{aligned}{} & {} 2 EA_f \left( c+t \right) ^2 \left( \gamma ^*_{,xx} - w_{,xxx} \right) - F \gamma ^* = 0 \nonumber \\{} & {} F \gamma ^*_{,x} - 2 EI_f w_{,xxxx} = { q_0(x) } \end{aligned}$$

It can be shown that the variable *F* is related to the shear stiffness of the core, it holds $F= GA_c (1+t/c)^2$ (note: $\kappa = 1$ holds since the shear stress is uniformly distributed). The angle $\gamma ^*$ may not be confused with the rotation angle of the core $\psi$, it holds $\gamma ^* = \gamma c/(c+t)$, see Fig. 11. Eliminating $\gamma ^*$ in (A1), one finds the sixth-order differential equations derived by Mead–Markus [11] and Di Taranto [13] (note: that the nomenclature is different in their original works)

A2 $$\begin{aligned}{} & {} \frac{ \partial ^6 w}{ \partial x^6} - F \left( \frac{ 1 }{2 EI_f} + \frac{ 1 }{ 2 EA_f (c+t)^2 } \right) w_{,xxxx} \nonumber \\{} & {} \quad = { -\frac{ 1 }{2 EI_f} q_{0,xx} + \frac{ F }{4 EI_f EA_f (c+t)^2} q_{0} } \end{aligned}$$

Eqs. (A1) and (A2) are equivalent, but Stamm and Witte’s notation is more convenient for implementation in symbolic software programs like MATHEMATICA, when boundary conditions are considered in terms of *w* and $\gamma ^*$. Furthermore, it is noted that the differential Eq. (A2) is also obtained if one neglects the bending stiffness of the core in Eq. (38). This demonstrates that the presented sandwich theory is a consistent generalization of the works by Mead–Markus and Di Taranto, who both neglect the longitudinal stiffness of the core from the beginning.

Solving the eigenvalue problem, one finds 4 trivial eigenvalues and 2 nontrivial ones

A3 $$\begin{aligned} \lambda _{1,2}= & {} \pm \sqrt{ \frac{ F \left( EI_f + EA_f \left( c+t \right) ^2 \right) }{ 2 \left( c+t \right) ^2 EI_f EA_f } } \nonumber \\{} & {} \quad \lambda _{3,4,5,6} = 0 \end{aligned}$$

hence the solution for the deflection reads with Eq. (A3)

A4 $$\begin{aligned} w(x) = W_1 + W_2 x + W_3 x^2 + W_4 x^3 + W_5 \textrm{exp} (\lambda _1 x) + W_6 \textrm{exp} (\lambda _2 x) \end{aligned}$$

It is noted that the eigenvalues Eqs. (A3) and (40) are identical if $EI_c \rightarrow 0$ holds.

### A.2 Fourth-order sandwich theory

Assuming a small thickness of the faces, the bending stiffness becomes negligible $EI_f \ll EA_f (c+t)^2$. Hence, one finds from Eq. (A1)

A5 $$\begin{aligned} 2 EA_f \left( c+t \right) ^2 \left( \gamma ^*_{,xx} - w_{,xxx} \right) - F \gamma ^* = 0 \nonumber \\ F \gamma ^*_{,x} = { q_0(x) } \end{aligned}$$

or from (A2)

A6 $$\begin{aligned} { -F w_{,xxxx} = -q_{0,xx} + \frac{ F }{2 EA_f (c+t)^2} q_{0} } \end{aligned}$$

The term $2 EA_f (c+t)^2$ (Steiner’s parallel axis theorem) can be interpreted as bending stiffness. Introducing $w = w_B + w_S$ one is able to split the deflection into two terms

A7 $$\begin{aligned} 2 EA_f (c+t)^2 w_{B,xxxx} = { q_0(x) } \nonumber \\ F w_{S,xxxx} = { q_{0,xx}(x) } \end{aligned}$$

This is also called the method of partial deflections, where $w_B$ is the deflection due to bending and $w_S$ is the deflection due to shear.

## Appendix B Simplified beam modeling

In the following, the main equations of the equivalent single layer model considering finite shear stiffness (denoted as $ESL_{{Timo}}$ and $BE_{{face}}$) are given. As already stated in Sect. 4, the author intends to make clear that none of these two models should be used when modeling typical sandwich beams. As the parameter variations of the core stiffness show (Figs. 5, 8 and 10) these models should be considered as limit cases only: For hard cores when $\mu \gg 1$ holds, $ESL_{{Timo}}$ will yield accurate results (Fig. 12a). For very soft cores when $\mu < 10^{-5}$ holds and the shear and bending stiffness of the core is rather low, $BE_{{face}}$ might be a good approximation (Fig. 12b).

## Appendix C $ESL_{Timo}$ model: equivalent single layer theory

Using the Timoshenko assumption for the sandwich cross section (cross sections remain plane, i.e., $u(x,z) = z \psi$), the governing equations follow from Eqs. (11) and (12)

C8 $$\begin{aligned}{} & {} \textrm{EI}_\textrm{ESL} {\psi }_{,xx} - \textrm{GA}^*_\textrm{ESL} \left( {\psi } + {w}_{,x} \right) = 0 \end{aligned}$$

C9 $$\begin{aligned}{} & {} \textrm{GA}^*_\textrm{ESL} \left( {\psi }_{,x} + {w}_{,xx} \right) - q_{0} = 0 \end{aligned}$$

where the shear and bending stiffness read

C10 $$\begin{aligned} \textrm{GA}^*_\textrm{ESL}= & {} 2 \left( G_c b c + 2 G_f bt \right) \kappa \end{aligned}$$

C11 $$\begin{aligned} \textrm{EI}_\textrm{ESL}= & {} \frac{2}{3} E_c bc^3 + \frac{2}{3} E_f b \left[ (c+2t)^3 - c^3 \right] \end{aligned}$$

## Appendix D $BE_{face}$ model: vanishing core elasticity

If the shear and bending stiffness of the core is very small (but the core is still incompressible), the shear stress in the core and at the interface will vanish. In case of identical external layers the governing equations for the two face layers (perfectly slipping) read

D12 $$\begin{aligned} 2 \textrm{EI}_{f} {w}_{,xxxx} = - q_0 \end{aligned}$$

Note this equation also follows from Eqs. (30) and (31): differentiating (31) with respect to *x* and setting $\textrm{GA}^*_{c} \rightarrow 0$ and $\textrm{EI}_c \rightarrow 0$, one finds

D13 $$\begin{aligned} \psi _{,xx} = \frac{t}{c} w_{,xxx} \end{aligned}$$

Equation (D12) follows by inserting the kinematic relation (D13) into Eq. (30).
